# Mesangial Cells Exhibit Features of Antigen-Presenting Cells and Activate CD4+ T Cell Responses

**DOI:** 10.1155/2019/2121849

**Published:** 2019-06-17

**Authors:** Hongyu Yu, Shaoyuan Cui, Yan Mei, Qinggang Li, Lingling Wu, Shuwei Duan, Guangyan Cai, Hanyu Zhu, Bo Fu, Li Zhang, Zhe Feng, Xiangmei Chen

**Affiliations:** ^1^Department of Nephrology, The Second Hospital of Jilin University, Changchun, Jilin, China; ^2^Department of Nephrology, Chinese PLA General Hospital, Chinese PLA Institute of Nephrology, State Key Laboratory of Kidney Diseases, National Clinical Research Center for Kidney Diseases, Beijing, China

## Abstract

**Background:**

Mesangial cells play a prominent role in the development of inflammatory diseases and autoimmune disorders of the kidney. Mesangial cells perform the essential functions of helping to ensure that the glomerular structure is stable and regulating capillary flow, and activated mesangial cells acquire proinflammatory activities. We investigated whether activated mesangial cells display immune properties and control the development of T cell immunity.

**Methods:**

Flow cytometry analysis was used to study the expression of antigen-presenting cell surface markers and costimulatory molecules in mesangial cells. CD4+ T cell activation induced by mesangial cells was detected in terms of T cell proliferation and cytokine production.

**Results:**

IFN-*γ*-treated mesangial cells express membrane proteins involved in antigen presentation and T cell activation, including MHC-II, ICAM-1, CD40, and CD80. This finding suggests that activated mesangial cells can take up and present antigenic peptides to initiate CD4+ T cell responses and thus act as nonprofessional antigen-presenting cells. Polarization of naïve CD4+ T cells (Th0 cells) towards the Th1 phenotype was induced by coculture with activated mesangial cells, and the resulting Th1 cells showed increased mRNA and protein expression of inflammation-associated genes.

**Conclusion:**

Mesangial cells can present antigen and modulate CD4+ T lymphocyte proliferation and differentiation. Interactions between mesangial cells and T cells are essential for sustaining the inflammatory response in a variety of glomerulonephritides. Therefore, mesangial cells might participate in immune function in the kidney.

## 1. Introduction

Mesangial cell-mediated glomerulonephritis (GN) is a frequent cause of end-stage renal disease (ESRD) [1, 2]. Mesangial cell injury is involved in the pathogenesis of IgA nephropathy (IgAN), diabetic nephropathy (DN), and lupus nephritis (LN) and plays an important role in the progression of kidney disease. The functions of mesangial cells include the formation of capillary loops during development, interactions with other renal cells, contractions to regulate capillary flow, and the removal of macromolecules. In addition to these actions, mesangial cells also play a role in promoting kidney inflammation [3]. Activated mesangial cells accumulate in injured mesangial areas, where they express cell adhesion molecules and secrete various proinflammatory cytokines [4–6].

Inflammatory cells are involved in the development of kidney disease [7, 8], but the local immunity in a diseased kidney is not completely understood. In some kidney diseases, a number of antigens are processed by antigen-presenting cells (APCs), such as dendritic cells (DCs) and B lymphocytes, and presented to T lymphocytes, which become activated and accumulate, leading to progressive inflammation of the kidney [9]. However, CD103+ kidney DCs can protect against progressive GN by maintaining IL-10-producing T regulatory (Treg) cells [10]. Therefore, APCs can play an important role in the resolution or progression of renal disease. Activated mesangial cells express molecules required for antigen presentation, such as major histocompatibility complex (MHC) class II, and might participate in local inflammatory responses by meeting the accessory cell requirement for the interaction with CD4+ T cells [11]. An animal model showed that activated CD4+ T cells target mesangial antigens and initiate GN [12, 13]. However, it is unclear whether these antigen-presenting features of mesangial cells impact CD4+ T cells.

We hypothesized that activated mesangial cells play a role in renal immune function, and in this study, we demonstrated that mesangial cells act as nonprofessional APCs to activate CD4+ T cells.

## 2. Materials and Methods

### 2.1. Cell Culture

Human primary mesangial cells (HMCs, ScienCell Research Laboratories Inc., Basel, Switzerland) were cultured in Mesangial Cell Medium (ScienCell Research Laboratories Inc.). Mouse mesangial cells (MMCs; ATCC, Manassas, VA, USA) were derived from glomerular explants of SV40 transgenic mice on the C57BL/6 background [14] and were cultured in Dulbecco's modified Eagle's medium/Ham's F12 medium (3 : 1 mixture) (ATCC) with 5% fetal bovine serum (FBS) (HyClone Laboratories Inc., South Logan, UT, USA). JAWSII immature mouse DCs (originating from the C57BL/6 mouse strain) (ATCC) were cultured in Alpha Minimum Essential Medium with 20% FBS and 5 ng/ml murine GM-CSF (PeproTech, Rocky Hill, NJ, USA) [15]. All the cells were grown in a humidified atmosphere (5% CO_2_) at 37°C. The HMCs were stimulated with 50 ng/ml human IFN-*γ* (Sigma-Aldrich, St. Louis, MO, USA) for 48 h [16]. The MMCs were stimulated with 50 ng/ml recombinant mouse IFN-*γ* (Sigma-Aldrich) for 48 h, and the JAWSII cells were stimulated with 2 *μ*g/ml lipopolysaccharide (LPS) (from *Escherichia coli* O111:B4, Sigma-Aldrich) for 48 h.

### 2.2. Isolation of Naïve CD4+ T Cells

Human naïve T cells, defined as CD4+ and CD45RA+ cells, were isolated from human peripheral blood mononuclear cells (PBMCs) through negative selection using a Naïve CD4+ T Cell Isolation Kit II (human) following the manufacturer's instructions. Briefly, naïve CD4+ T cells were negatively isolated with Naïve CD4+ T Cell Biotin-Antibody Cocktail II (biotin-conjugated monoclonal antibodies against CD8, CD14, CD15, CD16, CD19, CD25, CD34, CD36, CD45RO, CD56, CD123, TCR*γ*/*δ*, HLA-DR, and CD235a (Glycophorin A)) and Naïve CD4+ T Cell MicroBead Cocktail II using an LD column (Miltenyi Biotec) in the magnetic field of a suitable MACS separator. The flow-through containing unlabelled cells, which represented the enriched naïve CD4+ T cells, was collected (Figure S1(a)). Murine naïve CD4+ T cells, defined as CD45+, CD3*ε*+, CD4+, and CD62L+ cells, were isolated from the spleens of OT-II transgenic C57BL/6 mice (Jackson Laboratory, Bar Harbour, ME, USA) by negative selection using a Naïve CD4+ T Cell Isolation Kit (mouse) (Miltenyi Biotec, Auburn, CA) [17]. Briefly, naïve CD4+ T cells were negatively isolated with Biotin-Antibody Cocktail (biotin-conjugated monoclonal antibodies against CD8a, CD11b, CD11c, CD19, CD25, CD45R (B220), CD49b (DX5), CD105, MHC class II, Ter-119, and TCR*γ*/*δ*), anti-biotin MicroBeads, and CD44 MicroBeads using an LD column (Miltenyi Biotec) in the magnetic field of a suitable MACS separator. The flow-through containing unlabelled cells, which represented the enriched naïve CD4+ T cells, was collected (Figure S1(b)).

### 2.3. Coculture of Activated HMCs with Naïve CD4+ T Lymphocytes

Human PBMCs were isolated from normal volunteers. HMCs were stimulated with IFN-*γ* for 48 h, washed twice with PBS, trypsinized, and irradiated with 6,000 rads, which is a dose that has been shown to stop proliferation without affecting cell viability or membrane protein expression. Subsequently, the IFN-*γ*-treated HMCs were cocultured with freshly isolated naïve CD4+ T cells in RPMI 1640 (Gibco, Paisley, UK) with 10% FBS for 48 h. All of the procedures met the ethical guidelines, and the protocol was approved by the Hospital Research Ethics Committee.

### 2.4. Differentiation of Th1 Cells Cocultured with HMCs

One day before, a culture dish coated with anti-CD3*ε* antibodies (2 *μ*g/ml in PBS) was prepared. Naïve CD4+ T cells were cultured in Th1-differentiating culture medium (0.5 *μ*g/ml anti-CD28 antibody, 1 *μ*g/ml anti-IL-4 antibody, 5 ng/ml IL-2, and 10 ng/ml IL-12 in RPMI 1640 supplemented with 10% FBS, NEAA, antibiotics, and 55 *μ*M *β*-mercaptoethanol) (all from R&D Systems, Minneapolis, MN, USA) for 96 h according to the Th1 differentiation protocol [18]. Cells were plated in single wells of anti-CD3*ε*-coated 24-well culture dishes for 96 h. The cells were then washed twice with PBS and cocultured with HMCs for 48 h.

### 2.5. Flow Cytometry (FCM) Analysis

For the staining of cell surface molecules, the cells were suspended in staining buffer (FBS) (BD Pharmingen, San Diego, CA, USA) and stained with saturating concentrations of antibodies against HLA-DR, human CD40, human CD80, human ICAM-1, and mouse MHC class II (Miltenyi Biotec). The proportions of Th1 cells (IFN-*γ*+), Th2 cells (IL-4+), Th17 cells (IL-17+), and Treg cells (CD25+ and FOXP3+) were determined by FCM. The cells were activated with 50 ng/ml phorbol-12-myristate-13-acetate (PMA, Sigma-Aldrich), 250 ng/ml ionomycin (Sigma-Aldrich), and 1 *μ*g/ml Brefeldin A (BD Pharmingen) for 4 h at 37°C in a 5% CO_2_ atmosphere. The cells were then stained with antibodies against CD4 and CD25 (Miltenyi Biotec) for 15 min at room temperature. After cell fixation with 2% paraformaldehyde and permeabilization with 0.1% Triton X-100, intracellular antigens were stained with saturating concentrations of antibodies against IFN-*γ*, IL-4, IL-17, and FOXP3 (Miltenyi Biotec). FCM quantification of absolute cell numbers was performed using a Beckman Coulter flow cytometer (Miami, FL, USA).

### 2.6. Antigen-Processing Assays

To analyse the processing of soluble antigens, 40 *μ*g/ml DQ-ovalbumin (DQ-OVA) (Invitrogen) was used. HMCs were stimulated with or without 50 ng/ml IFN-*γ* for 48 h and then incubated with DQ-OVA for 24 h and 48 h at 37°C; the cells were then washed three times with ice-cold PBS, similar to the previously described protocol [19], and the cells were analysed via immunofluorescence and FCM [20].

### 2.7. OT-II Cell Stimulation Assays

For the stimulation assays, defined MMC populations treated with or without IFN-*γ* and LPS-treated DCs (JAWSII) were cultured for 2 days in the presence or absence of 1 mg/ml ovalbumin (OVA 323-339 peptide) (Sigma-Aldrich). The cells were intensively washed at least three times with PBS, and naïve CD4+ cells purified from OT-II mice were added at a ratio of 1 : 10. For the assessment of T lymphocyte DNA synthesis, an EdU solution was added 24 h prior to cell harvesting. After 48 h of coculture, the CD4+ cells were collected and evaluated using a Click-iT™ EdU Flow Cytometry Assay Kit (Invitrogen) according to the manufacturer's instructions. The CD4+ cells were analysed using a Beckman flow cytometer [21].

### 2.8. Real-Time PCR

Total RNA from mesangial cells or CD4+ T cells was isolated using the TRIzol Reagent (Invitrogen) according to the manufacturer's instructions. cDNA was synthesized with a ProtoScript II First-Strand cDNA Synthesis Kit (New England Biolabs (NEB), Beverly, MA, USA). RT-PCR was performed in triplicate using the SYBR Select Master Mix (Life Technologies, California, USA) and an Applied Biosystems 7500 Real-Time PCR system (ABI, Foster City, CA, USA). The threshold cycle (CT) values for target- and GAPDH-specific fragment amplification were determined with the ABI PRISM SDS7500 software, and the delta-CT values were calculated. Primers were used for the following genes (Table 1): HLA-DP, HLA-DQ, HLA-DR, ICAM-1, CD80, TGF-*β*, IL-1A, IL-4, IL-6, IL-12A, IL-17, IL-23A, CCL2, NF*κ*B, and GAPDH.

### 2.9. Western Blotting

The proteins from the cells were resolved on SDS-PAGE gels, transferred onto nitrocellulose membranes, and immunoblotted with antibodies against HLA-DR, ICAM-1, CD80, IL-1*α*, IL-6, NF*κ*B, CCL2, and *β*-actin (Abcam, Cambridge, MA, USA). Secondary antibodies were then applied, and the signals were detected using a ChemiDoc-It 600 Imaging System (UVP, Upland, USA).

### 2.10. ELISA

The levels of the secreted chemokines TGF-*β*, IFN-*γ*, IL-4, IL-6, IL-10, IL-12, IL-17, and IL-23 in culture supernatants were measured using Quantikine ELISA kits (R&D Systems) according to the manufacturer's recommended protocol. The absolute concentration of each sample was calculated based on the measured optical density of the sample using a standard curve.

### 2.11. Statistical Analysis

The results are expressed as the means and standard errors of the mean (SEMs). The statistical analyses were performed with Student's *t*-test or one-way analysis of variance (ANOVA) followed by Tukey's HSD post hoc test, and *P* values <0.05 were considered significant. The SPSS 19.0 software (IBM, Armonk, NY, USA) was used for the statistical analyses.

## 3. Results

### 3.1. Activated HMCs Express APC Surface Markers and Costimulatory Molecules

**Figure 1:**
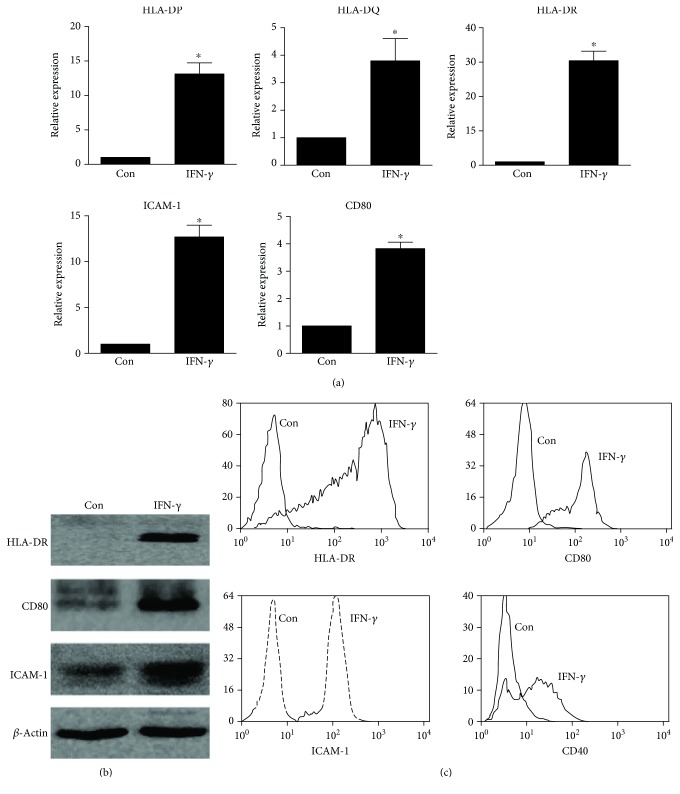
Expression of APC surface markers and costimulatory molecules in activated HMCs. (a) HMCs were incubated in medium containing IFN-*γ* (50 ng/ml) for 48 h, and the expression of HLA-DP, HLA-DQ, HLA-DR, ICAM-1, and CD80 was assessed by real-time PCR. (b) Western blots showing the protein expression levels of HLA-DR, ICAM-1, and CD80 in control and IFN-*γ*-treated HMCs after 48 h; *β*-actin was used as the loading control. (c) HMCs were cultured for 48 h with or without IFN-*γ* stimulation, and levels of the surface molecules HLA-DR, CD80, ICAM-1, and CD40 were determined by FCM. The data in (a) were analysed using Student's *t*-test. The data are representative of three independent experiments, and the error bars represent the means ± SEMs. Control (Con): HMCs treated without IFN-*γ*. ^∗^ *P* < 0.05 vs. Con.

Because APCs can mature, as indicated by the upregulation of characteristic surface molecules, we analysed their surface molecule expression. To test the response of HMCs to stimulation with IFN-*γ*, which is an important inflammatory cytokine that is mainly produced by effector T cells, HMCs were exposed to IFN-*γ* for 48 h of culture. HMCs expressed very low mRNA levels of the APC surface markers and the costimulatory molecules HLA-DP, HLA-DQ, HLA-DR, ICAM-1, and CD80. Exposure to IFN-*γ* markedly enhanced HLA-DP, HLA-DQ, HLA-DR, ICAM-1, and CD80 mRNA expression in HMCs (Figure 1(a)). A significant increase in MHC-II (HLA-DR), CD80, and ICAM-1 protein expression in the IFN-*γ*-treated HMCs was also observed via western blotting analysis compared with that in the controls (Figure 1(b)). Furthermore, we performed a flow cytometric analysis of HMC surface proteins. IFN-*γ*-treated HMCs expressed the antigen-presenting molecule MHC-II (HLA-DR) and costimulatory molecules, such as CD80, ICAM-1, and CD40, which are characteristic markers of professional APCs (Figure 1(c)). Collectively, these results show that HMCs express APC surface markers and costimulatory molecules.

### 3.2. Activated HMCs Have the Ability to Process Antigen In Vitro

**Figure 2:**
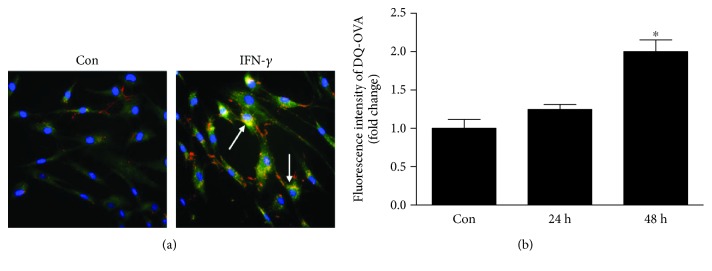
Activated HMCs process antigen. (a) HMCs were treated with or without IFN-*γ* for 48 h, incubated with DQ-OVA for 48 h at 37°C in medium, washed, fixed, and stained with DAPI. The arrows indicate DQ-OVA processing analysed via confocal microscopy. (b) HMCs were pretreated with IFN-*γ* or medium alone for 48 h, incubated for 24 and 48 h with fluorochrome-labelled ovalbumin (DQ-OVA), and analysed via flow cytometry. The data in (b) were analysed by one-way analysis of variance (ANOVA) followed by Tukey's HSD post hoc test. The data are representative of three independent experiments, and the error bars represent the means ± SEMs. Con: HMCs treated without IFN-*γ*. ^∗^ *P* < 0.05 vs. Con.

To further analyse the antigen-processing function of cells, we used DQ-OVA, a self-quenching conjugate that upon proteolysis produces fluorescent DQ-OVA-derived peptides that can be quantified using fluorescence-based techniques. We first examined the distribution of DQ-OVA in cells through confocal microscopy. The images showed that IFN-*γ*-treated HMCs exhibited stronger fluorescence signals than untreated HMCs (Figure 2(a)). The DQ-OVA peptide-associated fluorescence was also quantified by FCM. The intensity of DQ-OVA-stained HMCs treated with IFN-*γ* was markedly increased compared with that of untreated HMCs (Figure 2(b)). Taken together, these findings indicate that IFN-*γ*-treated HMCs are capable of processing antigen.

### 3.3. MMCs Activate Naïve CD4+ OT-II T Cells through MHC-II Presentation

**Figure 3:**
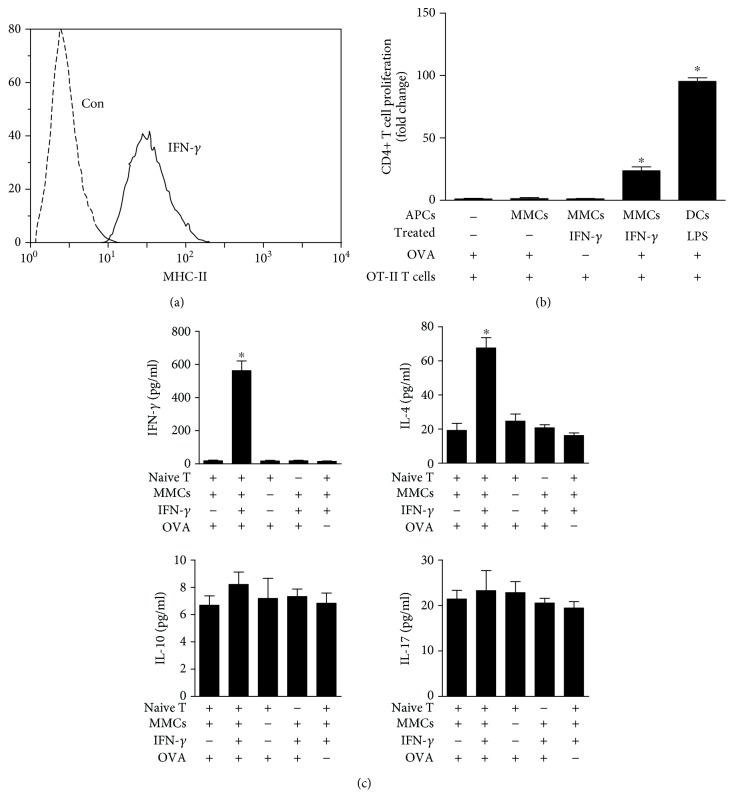
MMCs activate CD4+ T cells by MHC-II presentation. (a) Flow cytometric analysis of MHC-II molecules expressed on MMCs that were cultured for 48 h with IFN-*γ* stimulation. (b) IFN-*γ*-treated MMCs or LPS-treated DCs were incubated with or without ovalbumin (OVA, 1 mg/ml). Subsequently, antigen-pulsed MMCs or DCs were cocultured with naïve CD4+ T cells (OT-II). Additionally, OT-II T cell preparations were incubated with ovalbumin (1 mg/ml) alone. Two days after stimulation, antigen-specific proliferation was analysed with an EdU assay and detected by FCM. The results are expressed as fold changes in EdU-positive CD4+ T cells (OT-II). (c) MMCs were treated with IFN-*γ* for 48 h, extensively washed, irradiated, and cocultured with naïve CD4+ OT-II T cells and ovalbumin (1 mg/ml) for 48 h. Cytokine expression was analysed via ELISA to determine the expression of IFN-*γ*, IL-4, IL-10, and IL-17 in the culture supernatant. The data in (b) and (c) were analysed by one-way analysis of variance (ANOVA) followed by Tukey's HSD post hoc test. The error bars represent the means ± SEMs, and the graphs are representative of at least three independent experiments with similar results; ^∗^ *P* < 0.05.

We subsequently aimed to elucidate the antigen presentation function of mesangial cells with regard to CD4+ T cell stimulation. To this end, we isolated antigen-specific naïve CD4+ T cells from OT-II T cell receptor- (TCR-) transgenic mice. Because the naïve CD4+ OT-II T cells were isolated from mice, we used MMCs for coculture, and the naïve CD4+ OT-II T cells were cocultured with LPS-matured DCs as APCs. IFN-*γ*-treated MMCs expressed the antigen-presenting molecule MHC-II, which is a characteristic marker of professional APCs (Figure 3(a)). MMCs or DCs (JAWSII) were pulsed with ovalbumin (OVA), which is specifically recognized by CD4+ OT-II T cells, and subsequently cocultured with naïve CD4+ OT-II T cells. CD4+ OT-II T cells were specifically stimulated by IFN-*γ*-treated MMCs or LPS-treated DCs presenting the OVA peptide in the context of MHC-II. OVA-specific CD4 T cell proliferation was induced by IFN-*γ*-treated MMCs or LPS-treated DCs, and the direct pulsing of CD4+ T cells with OVA in the absence of IFN-*γ*-treated MMCs or LPS-treated DCs did not result in proliferation (Figure 3(b)). To determine the effect of IFN-*γ*-stimulated MMCs on the activation of naïve CD4+ T cells, we measured T cell cytokine markers, including IFN-*γ* (Th1 cells), IL-4 (Th2 cells), IL-10 (Th2 cells), and IL-17 (Th17 cells). The IFN-*γ* and IL-4 levels in the culture medium of CD4+ T cells activated by IFN-*γ*-stimulated MMCs were significantly increased compared with those found for the other groups. However, these CD4+ T cells produced very little IL-10 or IL-17 (Figure 3(c)). In conclusion, IFN-*γ*-treated MMCs represent professional APCs that can present peptides to CD4+ T cells and activate CD4+ T cell proliferation in vitro.

### 3.4. Activated HMCs Drive the Differentiation of Naïve CD4+ T Cells into Th1 Effectors

**Figure 4:**
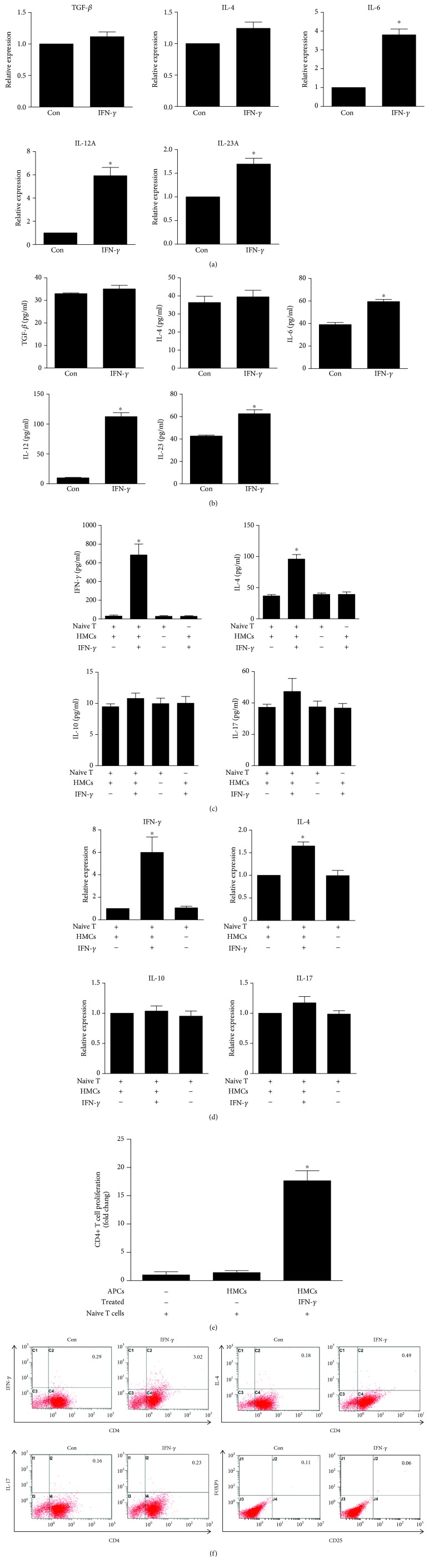
Activated HMCs induce the differentiation of naïve CD4+ T cells. (a) The mRNA expression of TGF-*β*, IL-4, IL-6, IL-12A, and IL-23A in HMCs treated with IFN-*γ* for 48 h was analysed by RT-PCR. (b) The protein expression of TGF-*β*, IL-4, IL-6, IL-12, and IL-23 in cell culture supernatants from HMCs treated with IFN-*γ* for 48 h was analysed via ELISA. (c) HMCs were treated with IFN-*γ* for 48 h, extensively washed, irradiated, and cocultured with naïve CD4+ T cells for 48 h. ELISA was used to determine the expression of the cytokines IFN-*γ*, IL-4, IL-10, and IL-17 in the culture supernatant. (d) HMCs were treated with IFN-*γ* for 48 h, extensively washed, irradiated, and cocultured with naïve CD4+ T cells for 48 h. The mRNA expression of IFN-*γ*, IL-4, IL-10, and IL-17 in CD4+ T cells was analysed via RT-PCR. (e) HMCs were treated with IFN-*γ* for 48 h, extensively washed, irradiated, and cocultured with naïve CD4+ T cells for 48 h. Two days after stimulation, proliferation was analysed using an EdU assay and detected by FCM. The results are expressed as fold changes in EdU-positive CD4+ T cells. (f) HMCs cultured alone or in the presence of IFN-*γ* were used to activate naïve CD4+ T cells. The differentiation of activated T cells into Th1 (IFN-*γ*+), Th2 (IL-4+), Th17 (IL-17+), or Treg (FOXP3+, CD25+) effectors was assessed via intracellular staining and flow cytometry after 48 h. The data in (a) and (b) were analysed by Student's *t*-test, and the data in (c), (d), and (e) were analysed by one-way analysis of variance (ANOVA) followed by Tukey's HSD post hoc test. The data are representative of three independent experiments, and the error bars represent the means ± SEMs. Con: HMCs treated without IFN-*γ*. ^∗^ *P* < 0.05.

To assess the effect of activated mesangial cells on T cells, we assayed the properties of T cells activated by IFN-*γ*-stimulated HMCs. First, we examined the cytokine expression levels of HMCs under basal and IFN-*γ*-stimulated conditions (Figure 4(a) for mRNA and Figure 4(b) for protein). HMC activation by IFN-*γ* for 48 h resulted in increases in IL-6, IL-12A, and IL-23A mRNA expression (Figure 4(a)). The IL-6, IL-12, and IL-23 protein levels in cell culture supernatants of IFN-*γ*-treated HMCs were significantly increased compared with those in supernatants of untreated HMCs (Figure 4(b)). However, there appeared to be no statistically significant difference between IFN-*γ*-treated HMCs and control cells with respect to the expression of TGF-*β* and IL-4 (Figures 4(a) and 4(b)). HMCs were activated by IFN-*γ* and subsequently cocultured with naïve CD4+ T cells for 48 h. To determine the effect of IFN-*γ*-stimulated HMCs on the activation of CD4+ T cells, we measured cytokine markers of T cells, including IFN-*γ*, IL-4, IL-10, and IL-17. High levels of IFN-*γ* were detected in the culture medium of CD4+ T cells activated by IFN-*γ*-stimulated HMCs. The IL-4 level in the culture medium of CD4+ T cells activated by IFN-*γ*-stimulated HMCs was found to be significantly increased compared with that in the other groups, but these CD4+ T cells produced very little IL-10 or IL-17 (Figure 4(c)). Quantitative real-time PCR demonstrated that the IFN-*γ* and IL-4 levels were increased significantly in CD4+ T cells cocultured with IFN-*γ*-stimulated HMCs compared with the levels observed in the control group (Figure 4(d)). CD4+ T cell proliferation was analysed using an EdU assay and detected by FCM, and CD4+ T cell proliferation was found to be induced by IFN-*γ*-treated HMCs (Figure 4(e)). Nontreated HMCs induced T cell populations that produced very little IFN-*γ*, IL-4, or IL-17, and IFN-*γ*-stimulated HMCs induced a significant percentage of IFN-*γ*-producing T cells and IL-4-producing T cells (Figure 4(f)). Coculture significantly increased the differentiation of IFN-*γ*+ Th1 and IL-4+ Th2 cells; therefore, this strategy effectively induces IFN-*γ*+ Th1 over IL-4+ Th2 cells. These data suggest that naïve CD4+ T cells activated by IFN-*γ*-treated HMCs predominantly undergo Th1 differentiation.

### 3.5. Th1 Cells Lead to HMC Activation

**Figure 5:**
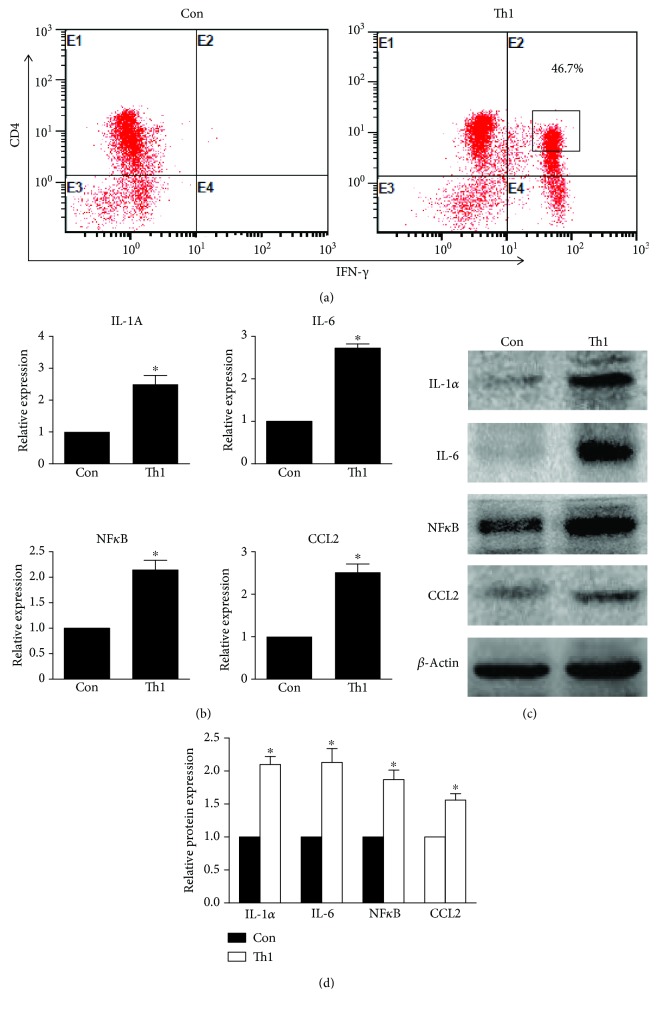
Th1 cells mediate proinflammatory effects in HMCs. (a) Naïve CD4+ T cells develop into Th1 cells after stimulation with anti-CD3*ε* antibody and Th1-differentiating culture medium. (b) Cytokine expression was analysed via RT-PCR, and the results revealed that Th1 cells induced markedly increased IL-1A, IL-6, CCL2, and NF*κ*B mRNA expression in HMCs. (c) Representative western blots showing the protein expression levels of IL-1*α*, IL-6, CCL2, and NF*κ*B in mesangial cells after interaction with Th1 cells for 48 h; *β*-actin was used as the loading control. (d) The relative quantitation of IL-1*α*, IL-6, CCL2, and NF*κ*B protein expression in each group was determined by western blot analysis; *β*-actin served as the internal reference. The data in (b) and (d) were analysed using Student's *t*-test. The data are representative of three independent experiments, and the error bars represent the means ± SEMs. Con: HMCs cocultured with Th0 cells. ^∗^ *P* < 0.05 vs. Con.

To further assess the effect of Th1 cells on mesangial cells, we detected the cytokine expression of HMCs that were cocultured with Th1 cells but not treated with IFN-*γ*. We used an anti-CD3*ε* antibody and Th1-differentiating culture medium to induce Th0 cell polarization to Th1 cells (Figure 5(a)) and then cocultured the T cells with mesangial cells for 48 h. The IL-1A, IL-6, CCL2, and NF*κ*B mRNA expression levels were increased significantly in the mesangial cells cocultured with Th1 cells compared with the levels observed in the control group (Figure 5(b)). We also found that the IL-1*α*, IL-6, CCL2, and NF*κ*B protein levels in HMCs cocultured with Th1 cells were increased compared with the levels observed in the control group (Figures 5(c) and 5(d)). These results demonstrate that Th1 cells lead to HMC activation.

## 4. Discussion

Mesangial cells have been recognized as an important factor in the pathogenesis of glomerular injury in many different glomerular diseases [22]. Mesangial cell activation induces distinct phenotypes in response to changes in the glomerular microenvironment [23]. Our study provides evidence showing that activated mesangial cells express the membrane proteins required for antigen presentation and modulate CD4+ T lymphocyte proliferation. Similar properties have been described in hepatic stellate cells and astrocytes, which indicates that under certain circumstances, these cells could play a supporting role in the local regulation of the immune system [24, 25]. Based on our findings, we propose that mesangial cells play a role in immune function in the kidney.

MHC-II molecules are surface glycoproteins that bind exogenous antigens and present them to TCRs and are considered important markers of APCs. The expression profile of MHC and costimulatory molecules on professional or nonprofessional APCs can greatly influence the magnitude of T cell activation. The Th1 cytokine IFN-*γ* alone or in combination with other cytokines has been reported to induce MHC class II expression in many different cell types [24, 26–28]. IFN-*γ* primarily enhances MHC-II expression by upregulating the expression of MHC class II transactivator (CIITA), a transcriptional coactivator that is essential for MHC-II expression [29]. The upregulation of the MHC class II gene by IFN-*γ* is a critical process in antigen presentation and leads to the activation of T cell antigen presentation and thereby activation of T cell-mediated immune reactions [30]. IFN-*γ* induces the expression of a set of early response genes through the formation of a ligand-dependent multimolecular complex containing IFN-*γ* receptor chains (a and b), Janus tyrosine kinases (Jak1 and Jak2), and the transcription factor Stat1a [31], which indicates that Stat1a is critical for CIITA and MHC class II expression in IFN-*γ*-induced mesangial cells.

HMCs stimulate CD4+ T cells to proliferate and synthesize cytokines since there is no nominal antigen introduced. IFN-*γ* stimulates human mesangial cells to secrete cytokines such as IL-6, IL-12A, and IL-23, which may stimulate T cell proliferation and cytokine secretion. A subset of human peripheral blood CD4+ T cells can be activated with the combination of IL-12 and IL-18 to produce IFN-*γ* in the absence of any antigenic stimulation [32], suggesting that naïve CD4+ T cells may participate in innate immunity or amplify adaptive immune responses through cytokine-induced antigen-independent cytokine production. In our experiment, human naïve CD4+ T cells were cocultured with mesangial cells that were not stimulated by IFN-*γ* and did not release cytokines or proliferate. These cytokines were produced only after IFN-*γ* stimulation of mesangial cells, indicating their potential specific involvement in the indirect interactions of IFN-*γ*-stimulated HMCs and CD4+ T cells. A limitation of the present study is that the HLA genotype of naïve human CD4+ T cells was not detected. The exact role of MHC molecules in the peripheral survival and proliferation of naïve T cells is controversial, as some studies have suggested that they are critically required, whereas others have suggested that they are not [33, 34]. In some cases, MHC molecules signal via the antigen-specific portions of the TCR to initiate productive immune responses, whereas in other cases, they may signal through other molecules, such as CD4 and CD2, which can occur independent of TCR signalling [35]. Numerous in vitro studies have demonstrated that T cells are dependent on cytokines for antigen-induced proliferation. The cell-cell interactions between T cell and antigen-presenting cells (APCs) have been extensively studied using the proliferation assay in which T cell populations from primed animals proliferate when reexposed to the priming antigen. Because human cells lack an ideal specific antigen for coculture, we selected mouse CD4+ T cells and mesangial cells from the C57BL/6 background and OVA antigen coculture to study whether mesangial cells have antigen-presenting functions. The current study has provided the groundwork for understanding the nature of the interactions (indirect and direct) between mesangial cells and T cells.

Previous studies have detected the expression of antigen presentation molecular markers in mesangial cells [11], but few have explored the APC function of these cells. Human and murine mesangial cells were used in our study. Mesangial cells from different species have similar effects and can be induced by IFN-*γ* to express antigen-presenting molecular markers and secrete cytokines. With the aim of elucidating the antigen presentation function of mesangial cells, mesangial cells presenting the OVA antigen to activate naïve CD4+ OT-II T cells were detected. The evidence for the APC function of mesangial cells relies on the coculture of mesangial cells, ovalbumin, and naïve CD4+ OT-II T cell preparations. Because the CD4+ OT-II cells were obtained from a C57BL/6 mouse source, we selected mouse mesangial cells for the coculture with the CD4+ OT-II cells. Activated mesangial cells processed the ovalbumin protein for antigen-specific CD4+ OT-II T cell stimulation. OT-II mice express *α*- and *β*-chains of the T cell receptor and CD4 coreceptors that are specific for recognizing chicken ovalbumin 323–339 presented by MHC-II molecules; thus, these mice are useful for in vitro and in vivo studies of T cell biology, such as those investigating TCR-ligand interactions, antigen presentation, and T cell activation [36]. Ovalbumin-specific CD4+ OT-II T cell activation is induced by renal tubular epithelial cells or podocytes, which act as nonprofessional APCs to trigger specific T cell responses in the kidney [37, 38].

Immune-mediated damage to glomerular structures is largely responsible for the pathology associated with the majority of glomerular diseases [39]. Genetically modified immune responses to infection and self-antigens initiate most forms of GN by generating pathogen- and danger-associated molecular patterns that stimulate Toll-like receptors and complement [40]. The presence of immune cells and their mediators in the glomerulus is a strong indicator of infection or a developing pathologic process. The innate immune responses activate circulating monocytes and resident glomerular cells to release inflammatory mediators and initiate adaptive, antigen-specific immune responses that collectively damage glomerular structures. All subsets of T cells, including CD4 helper cells of the Th1, Th2, and T regulatory (Treg) lineages, have been implicated in GN, and IL17-producing Th17 cells induce inflammation. Studies of experimental glomerulonephritis have shown that Th1 and Th17 cells contribute to glomerular damage [41]. Th2 cells play a role in glomerular disease by activating B cells, which produce antibodies that contribute to glomerular injury through their deposition as immune complexes in the glomerulus. A unique pathway termed cross-presentation allows DCs to present exogenous antigens via MHC class I molecules to CD8+ T cells. Following activation, CD8+ T cells differentiate into cytotoxic T cells that also play a role in glomerular disease. T cells are attracted through mechanisms involving chemokines and their receptors and release cytokines, such as IFN-*γ*, TNF-*α*, IL-4, and IL-17, which induce other cells to produce additional proinflammatory chemokines that activate resident glomerular cells, including mesangial cells. Mesangial cells play a critical role in the initiation of glomerular inflammation through their ability to detect and respond to immune complexes, aberrantly glycosylated IgA, oxidative stress, cytokines, chemokines, and lytic toxins, including the membrane attack complex. In response to these signals, mesangial cells produce inflammatory cytokines and chemokines that recruit and activate inflammatory cells [42, 43]. Once activated, macrophages and neutrophils perpetuate glomerular damage by releasing inflammatory cytokines and chemokines that recruit additional inflammatory cells into the glomerulus, and this effect activates mesangial cells, stimulates their dedifferentiation, and induces the release of growth factors that drive hypertrophy and mesangial expansion.

CD4+ T cells play an important role in mesangial proliferative glomerulonephritis (MsPGN) [44, 45]. CD4+ T cells that recognize specific antigens deposited in the glomerular mesangium cause glomerulonephritis-like kidney injury. CD4+ T cell-mediated inflammation induces mesangial cell activation and increases in glomerular MCP1 and fibronectin, which shows that T cells reactive to antigens in the mesangium are sufficient for the initiation of glomerular pathology [12]. Anti-CD5 mAb treatment suppresses CD4+ T cell recruitment into glomeruli and reduces proteinuria and mesangial injury [46]. Th1 cells mediate proinflammatory cellular immunity, and their ability to secrete IFN-*γ* and activate mesangial cells is important in the progression of MsPGN [47]. HMCs stimulated by IgA1 can produce CCL20 and consequently recruit inflammatory Th17 cells to the kidneys to induce further lesions in IgA nephropathy [43]. The immune balance towards the proinflammatory/Th1 phenotype in mesangial cells might initiate and/or prolong inflammation, resulting in glomerular disease. A previous study reported that high glucose stimulation increases proinflammatory/Th1 gene expression but decreases Th2-related gene expression in mesangial cells [48]. Patients with MsPGN have elevated serum IFN-*γ* levels and decreased serum IL-10 levels compared with those in healthy controls [49]. Increased intrarenal gene expression of proinflammatory and Th1 cytokines (IFN-*γ* and IL-2) is associated with glomerular lesions in IgAN [50]. The cytokine balance is fundamentally skewed towards a Th1 phenotype in MsPGN patients, and immunoregulatory factors counter this shift in the Th1/Th2 balance and thereby produce therapeutic effects [49, 51]. These results support the dominance of the contribution of the interaction between Th1 cells and mesangial cells to the pathogenesis of MsPGN.

## 5. Conclusions

In summary, we provide evidence showing that activated mesangial cells express the molecules necessary for antigen presentation and modulate lymphocyte proliferation. Local cell-to-cell interactions mediated by mesangial cells and T cells are essential for sustaining the inflammatory response in a variety of glomerulonephritides. Therefore, mesangial cells might participate in the immune function of the kidney.

## Figures and Tables

**Table 1:** List of primers used in reverse transcriptase-polymerase chain reaction.

Name	Sense primer (5′–3′)	Antisense primer (5′–3′)
HLA-DP	CACCAACCTGATCCGTAA	GACTGTGCCTTCCACTCC
HLA-DQ	TCTACCGCTGCTACCAAT	CCACAAGACAAATGAGGG
HLA-DR	GGCTTGAAGAATTTGGAC	TGATCGGAGTATAGTTGGA
ICAM-1	GCAAGAAGATAGCCAACCA	TGCCAGTTCCACCCGTTC
CD80	CCACCTTGCCCTTTACGT	GCCCACCATATTCCTCTA
TGF-*β*	TGTCACGGCAGCCGAATT	CCTGGAGCACCTGATAAACG
IL-1A	TGACGACGCACTTGTAGC	TCAGTCTTCTTCGCCTTT
IL-4	GCAGTTCCACAGGCACAA	TGGTTGGCTTCCTTCACA
IL-6	GGAGACTTGCCTGGTGAA	ACAGCTCTGGCTTGTTCC
IL-12A	CTCCAAACCGTTGTCATT	AATAGTCCCATCCTTCTTT
IL-17	CACCATGTGGCCTAAGAG	AGTCCGAAATGAGGCTGT
IL-23A	AGCCAGATTTGAGAAGAAG	GCAACAGCAGCATTACAG
CCL2	AGAATCACCAGCAGCAAG	GGAATCCTGAACCCACTT
NF*κ*B	ACTGGAAGCACGAATGAC	CAAATAGGCAAGGTCAGG
GAPDH	ACAACTTTGGTATCGTGGAA	CACAGTCTTCTGGGTGGC

## Data Availability

The data used to support the findings of this study are available from the corresponding author upon request.

## Supplementary Materials

Supplementary Materials Supplementary Figure 1 (S1): isolation of naïve CD4+ T cells by magnetic microbeads.
