# Supplementary figures and images for: Mesangial Cells Exhibit Features of Antigen-Presenting Cells and Activate CD4+ T Cell Responses
Source: J Immunol Res. 2019 Jun 17;2019:2121849. doi: 10.1155/2019/2121849 (PMC6604415; doi:10.1155/2019/2121849)

Figure S1

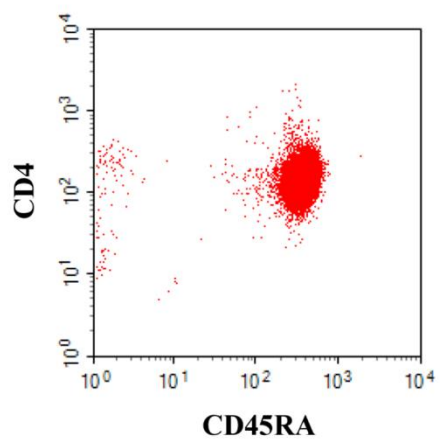

(a)

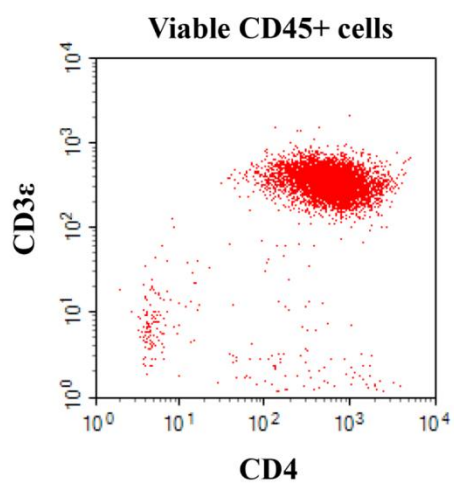

**Gated on CD45+ CD3 $\epsilon$ + CD4+ T cells**

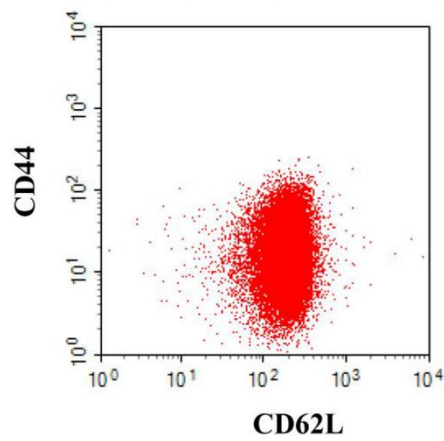

(b)

Supplement: Supplementary Materials — Supplementary Figure 1 (S1): isolation of naïve CD4+ T cells by magnetic microbeads. [file 2121849.f1.pdf]
